# The Relationship Between Subacute Pain, Chronic Pain, and Sleep Disorder: A Cross‐Sectional Study Based on NHANES (2009–2010)

**DOI:** 10.1002/brb3.70976

**Published:** 2025-10-21

**Authors:** Yanlin Yang, Gaohui Wu, Minxian Wang, Shuai Zhao

**Affiliations:** ^1^ Department of Anesthesiology Zhongnan Hospital of Wuhan University Wuhan China; ^2^ Department of Anesthesiology The Central Hospital of Wuhan Wuhan China; ^3^ Department of Internal Medicine Zhongnan Hospital of Wuhan University Wuhan China

**Keywords:** chronic pain, NHANES, sleep disorder, subacute pain

## Abstract

**Background:**

Chronic pain (CP) and subacute pain (SAP) represent major public health challenges, frequently coexisting with sleep disorders (SD). However, the association between pain duration and SD remains poorly characterized.

**Methods:**

This cross‐sectional study analyzed data from the National Health and Nutrition Examination Survey (NHANES, 2009–2010). CP, SAP, and SD were assessed through structured interviews conducted by trained personnel using the Computer‐Assisted Personal Interviewing (CAPI) system. Multivariable logistic regression models were employed to evaluate the relationship between pain duration (CP vs. SAP) and SD, while subgroup analyses explored potential effect modifications by analgesic use and pain intensity.

**Results:**

Among 1,109 participants, after adjusting for confounders, individuals with CP exhibited an 85% higher likelihood of SD compared to those with SAP (OR = 1.85, 95% CI: 1.39–2.47, *p* < 0.001). Pain that worsened during the day (OR = 1.95, 95% CI: 1.35–2.80, *p* < 0.001), persisted at rest (OR = 2.38, 95% CI: 1.65–3.43, *p* < 0.001), failed to be alleviated by exercise (OR = 1.59, 95% CI: 1.20–2.11, *p* = 0.001), and awakened from (OR = 2.70, 95% CI: 2.06–3.55, *p* < 0.001) was significantly associated with SD. Subgroup analyses revealed no significant interaction effects of analgesic use or pain intensity on the CP/SAP‐SD association (*p* for interaction > 0.05).

**Conclusion:**

These findings suggest that pain (both subacute and chronic) may be an independent risk factor for SD, supporting the need for early intervention for SAP.

## Graphical abstract

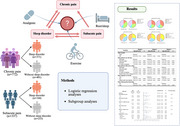

## 1 Introduction

Subacute pain (SAP) refers to pain persisting for 6–12 weeks, while chronic pain (CP) denotes pain continuing for more than 12 weeks (Banerjee and Argaez 2019). SAP may progress to CP in the absence of appropriate clinical intervention. According to a study by the U.S. Centers for Disease Control and Prevention (CDC), the estimated prevalence of CP is 20.4% in the general population (Dahlhamer et al. 2018). CP constitutes a widespread and significant global health challenge, imposing a substantial public health burden. SD is highly prevalent among U.S. adults. Data from the NHANES sleep disorders questionnaire indicate that approximately 29.8% of adults report sleep difficulties, while 27.2% experience excessive daytime sleepiness (Di et al. 2022). A reduction in SD demonstrates a strong association with both the prevalence of cardiovascular events and depressive disorders (Covassin and Somers 2023, Olfati et al. 2024). Existing research indicates a bidirectional relationship between pain and SD. Accordingly, the use of nalgesics in individuals with chronic pain can lead to modest improvements in sleep quality (Tang et al. 2019). This interaction relationship may be mediated through central sensitization mechanisms, whereby SD contributes to enhanced pain perception via neurological hyperexcitability (Herrero Babiloni et al. 2020, Krause et al. 2019). The interplay between pain duration and SD remains underexplored in the general population.

Previous studies have primarily examined the association between SD and CP within specific anatomical regions, without adequately accounting for the potential comorbidity of multisite pain. This oversight constitutes a significant methodological limitation, as the relationship between localized pain and SD may be confounded by both the spatial distribution and intensity of concurrent pain (Zhong and Wang 2024, Tong et al. 2024). Substantial evidence has investigated the bidirectional association between CP and SD (Haack et al. 2020, Chang et al. 2022, Runge et al. 2024). However, no study has yet examined the gradient relationship between pain duration (subacute/chronic) and SD using population‐based data from NAHNES.

This study had two primary objectives: to explore the association between SAP/CP and SD, and to examine potential effect modification by pain intensity and analgesic use. The hypothesis suggests that CP would exhibit a stronger association with SD compared to SAP, independent of analgesic medications and pain intensity.

## 2 Methods

### 2.1 Study Population and Data Source

**FIGURE 1:**
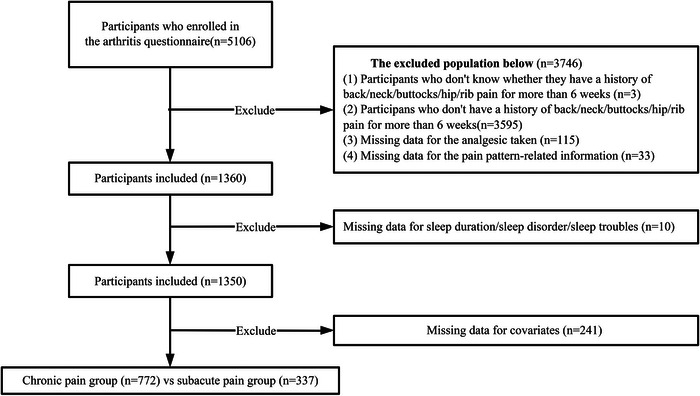
Flowchart of participants’ selection.

This cross‐sectional study utilized publicly available data from NHANES (https://www.cdc.gov/nchs/nhanes/index.html), a nationally representative database documenting the health and nutritional status of U.S. civilians. All participants provided written informed consent before enrollment. The study population was aged between 20 and 69 years from the 2009–2010 NHANES cycle who completed the arthritis questionnaire (ARQ was only administered during the 2009–2010 cycle and was not included in other survey years between 1999 and 2023). Initial screening identified 5,106 eligible participants, who were queried regarding persistent pain (≥ 6 weeks) in the neck, back, hips, or ribs. SD status was ascertained from the sleep disorders questionnaire (SLQ). The participants who have no history of pain for more than 6 weeks (n = 3598), missing data for analgesic taken/pain pattern‐related information (n = 148), incomplete information about sleep duration, SD, sleep troubles (n = 10), and populations with missing covariate data (n = 241) were excluded. After exclusions, the analytical sample comprised the SAP group (pain duration: 6 weeks–3 months; n = 337) and the CP group (pain duration: ≥ 3 months; n = 772) (Figure 1).

### 2.2 Pain‐Related Information

**FIGURE 2:**
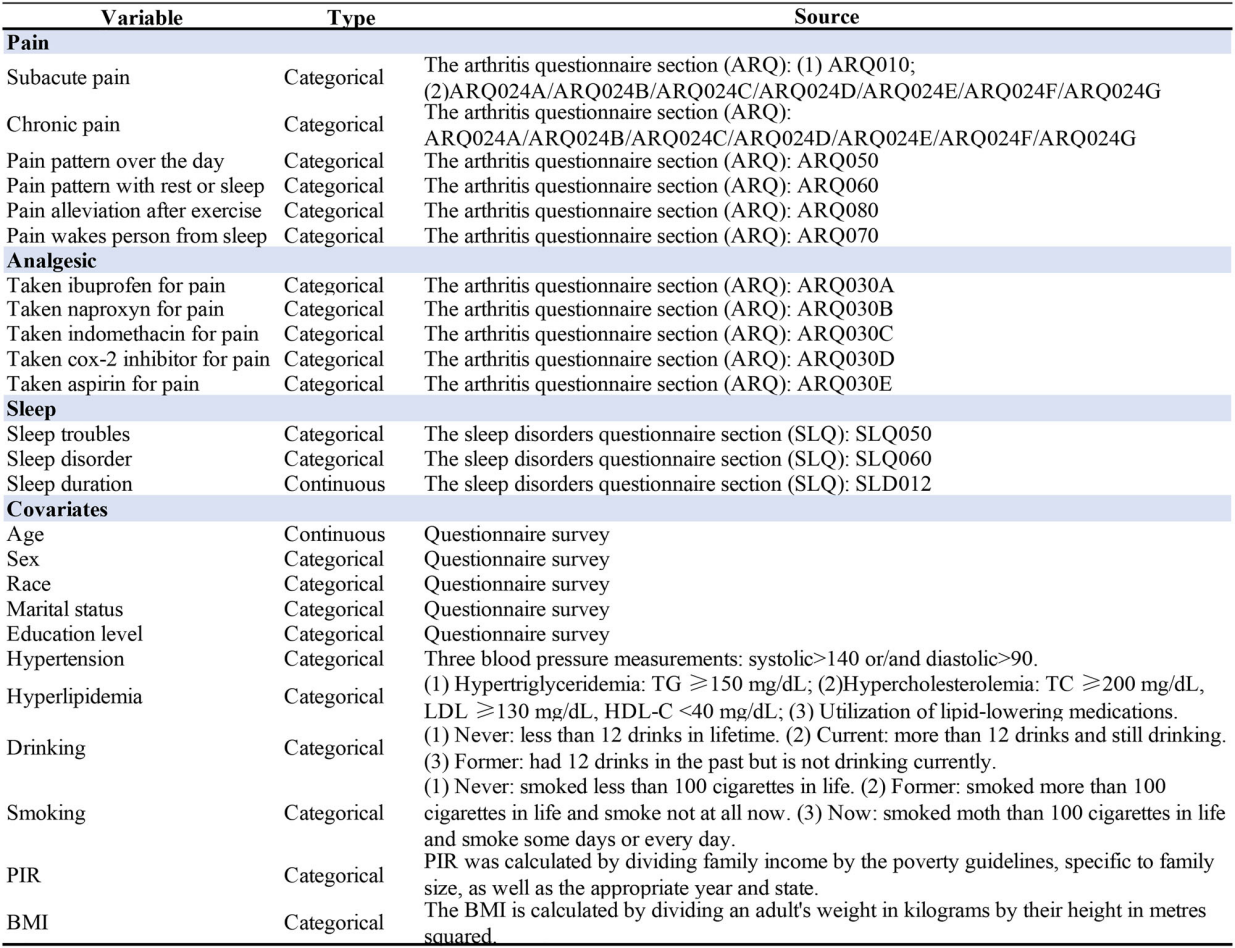
The definition of variables. Abbreviations: BMI = body mass index; PIR = family income to poverty ratio.

SAP is defined as pain in the back, neck, buttocks, hip, or rib that persists for a duration of 6 weeks to 3 months; chronic pain is described as pain in these areas that endures for more than 3 months. Information regarding analgesics derived from the inquiry: “For your back/neck/buttocks pain, aching, or stiffness, have you ever taken any of the following medicines (including ibuprofen, naproxen, indomethacin, Cox‐2 inhibitor, and aspirin)?” The questionnaire inquired about trends in pain throughout the day or at rest, and any alleviation experienced through walking or stretching for 30 min (Figure 2). Information for all variables is available to the public on the official NHANES website.

### 2.3 Sleep‐Related Information

Sleep‐related data were obtained from the 2009–2010 NHANES questionnaire data. Sleep duration was assessed using the survey item: “How much sleep do you usually get at night on weekdays or workdays?” Responses were analyzed as a continuous variable and subsequently categorized into three groups based on prior publication (Khosla and Lowe 1967, Andersen et al. 2018). The presence of SD was determined using two diagnostic screening questions: (Banerjee and Argaez 2019) “Have you ever been told by a doctor or other health professional that you have a sleep disorder?”, and (Dahlhamer et al. 2018) “Have you ever been told by a doctor or other health professional that you have trouble sleeping?” Participants who responded “yes” to both questions were classified as having SD, whereas those who answered “no” or “don't know” to either question were categorized as non‐SD.

### 2.4 Covariates

The covariates were chosen based on previous research (Di et al. 2022, Li and Shang 2021, Xu et al. 2022). The following covariates may influence the association between SAP, CP, and SD: sex, age, race (white, black, Mexican, or others), education level (less than high school, high school, or more), marital status (married/living with a partner or widowed/divorced/separated/never married), poverty‐income ratio (PIR, the threshold of poverty is set as 1), and body mass index (BMI, underweight, < 18.5 kg/m^2^; normal, 18.5–25.0 kg/m^2^; overweight, 25.0–30.0 kg/m^2^; obese, ≥ 30.0 kg/m^2^) (Clinical guidelines on the identification 1998, Khosla and Lowe 1967). The criteria for hypertension, hyperlipidemia, smoking status, and alcohol status are presented in Figure 2.

### 2.5 Statistical Analysis

The mean (Q1, Q3) was described for continuous variables with skewed distributions. Categorical variables were described as percentages. Based on the distribution's normality, the Wilcoxon and chi‐squared tests are employed to examine whether the risk of SAP and CP differs by sociodemographic and health characteristics. Multivariate logistic regression was employed to elucidate the association between pain‐related variables and the incidence of SD. Three logistic regression models were constructed to adjust for potential confounders. Model 1 was not adjusted; Model 2 was adjusted for sex, age, and race; and Model 3 was adjusted for sex, age, race, marital status, education level, hypertension, hyperlipidemia, alcohol status, smoking status, PIR, and BMI. All data were analyzed using R (Version 4.3.1, The R Foundation, Vienna, Austria) and GraphPad Prism (Version 10.0.0, San Diego, California, USA). Statistical significance is indicated by a *p* value < 0.05.

## 3 Results

### 3.1 The Traits of Participants

After applying the inclusion and exclusion criteria, 1109 participants were involved in this research (Figure 1). The traits of all participants were shown in Table 1. Among 1,109 participants, 337 (30.4%) were in the SAP group and 772 (69.6%) in the CP group. The CP group was older (median age 48.0 [IQR 36.0–58.0] years vs 41.0 [30.0–52.0] years; *p* < 0.001), with higher proportions of former smokers in the CP group (26.42% vs 17.51%) and hypertension (44.30% vs 34.72%; *p* = 0.003), also had shorter median sleep duration (6.0 [6.0–8.0] hours vs 7.0 [6.0–8.0] hours; *p* = 0.013), and had higher rates of SD (48.06% vs 30.86%; *p* < 0.001) and waking from pain (57.64% vs 35.01%; *p* < 0.001). Differences were observed in analgesic use: ibuprofen (78.76% vs 71.22%; *p* = 0.006), naproxen (52.33% vs 40.06%; *p* < 0.001), indomethacin (2.98% vs 0.89%; *p* = 0.034), and Cox2‐inhibitor (17.49% vs 7.12%; *p* < 0.001). Furthermore, the CP group had higher proportions of multi‐analgesics taken (61.53% vs 47.77%; *p* < 0.001). Pain patterns over the day differed between the SAP and CP groups: increase (33.42% vs 15.73%; *p* < 0.001) and decrease (18.52% vs 33.83%; *p* < 0.001).

**TABLE 1:** The characteristics of participants in SAP and CP groups, NHANES 2009–2010.

Variables	Total (n = 1109)	SAP (n = 337)	CP (n = 772)	*P*
**Age, M (Q_1_, Q_3_)**	46.00 (34.00, 57.00)	41.00 (30.00, 52.00)	48.00 (36.00, 58.00)	< 0.001
**Sex, n (%)**				0.391
Male	505 (45.54)	160 (47.48)	345 (44.69)	
Female	604 (54.46)	177 (52.52)	427 (55.31)	
**Race, n (%)**				0.012
White	607 (54.73)	172 (51.04)	435 (56.35)	
Black	160 (14.43)	41 (12.17)	119 (15.41)	
Mexican	200 (18.03)	79 (23.44)	121 (15.67)	
Others	142 (12.80)	45 (13.35)	97 (12.56)	
**Marital status, n (%)**				0.365
Married/living with partner	682 (61.50)	214 (63.50)	468 (60.62)	
Widowed/divorced/separated/never married	427 (38.50)	123 (36.50)	304 (39.38)	
**Education level, n (%)**				0.781
< High school	277 (24.98)	80 (23.74)	197 (25.52)	
Completed high school	285 (25.70)	90 (26.71)	195 (25.26)	
> High school	547 (49.32)	167 (49.55)	380 (49.22)	
**PIR, n (%)**				0.399
< 1.00	310 (27.95)	100 (29.67)	210 (27.20)	
≥ 1.00	799 (72.05)	237 (70.33)	562 (72.80)	
**BMI, n (%)**				0.568
< 18.5 kg/m2	15 (1.35)	5 (1.48)	10 (1.30)	
18.5 to < 25.0 kg/m2	252 (22.72)	85 (25.22)	167 (21.63)	
25.0 to < 30.0 kg/m2	354 (31.92)	101 (29.97)	253 (32.77)	
≥ 30.0 kg/m2	488 (44.00)	146 (43.32)	342 (44.30)	
**Alcohol status, n (%)**				0.052
No	276 (24.89)	71 (21.07)	205 (26.55)	
Yes	833 (75.11)	266 (78.93)	567 (73.45)	
**Smoking status, n (%)**				< 0.001
Former	263 (23.72)	59 (17.51)	204 (26.42)	
Now	364 (32.82)	105 (31.16)	259 (33.55)	
Never	482 (43.46)	173 (51.34)	309 (40.03)	
**Hypertension, n (%)**				0.003
No	650 (58.61)	220 (65.28)	430 (55.70)	
Yes	459 (41.39)	117 (34.72)	342 (44.30)	
**Hyperlipidemia, n (%)**				0.895
No	267 (24.08)	82 (24.33)	185 (23.96)	
Yes	842 (75.92)	255 (75.67)	587 (76.04)	
**Pain pattern over the day, n (%)**				< 0.001
Decreases	257 (23.17)	114 (33.83)	143 (18.52)	
Stays the same	437 (39.40)	131 (38.87)	306 (39.64)	
Increases	311 (28.04)	53 (15.73)	258 (33.42)	
It varies, no pattern	104 (9.38)	39 (11.57)	65 (8.42)	
**Pain pattern with rest or sleep, n (%)**				< 0.001
Decreases	291 (26.24)	118 (35.01)	173 (22.41)	
Stays the same	434 (39.13)	129 (38.28)	305 (39.51)	
Increases	267 (24.08)	48 (14.24)	219 (28.37)	
It varies, no pattern	86 (7.75)	23 (6.82)	63 (8.16)	
Doesn't have rest or sleep pain	31 (2.80)	19 (5.64)	12 (1.55)	
**Pain wakes person from sleep, n (%)**				< 0.001
No	546 (49.23)	219 (64.99)	327 (42.36)	
Yes	563 (50.77)	118 (35.01)	445 (57.64)	
**Pain alleviation after exercise, n (%)**				0.162
No	316 (28.49)	83 (24.63)	233 (30.18)	
Yes	746 (67.27)	238 (70.62)	508 (65.80)	
Does not do these activities	47 (4.24)	16 (4.75)	31 (4.02)	
**Taken ibuprofen for pain, n (%)**				0.006
No	261 (23.53)	97 (28.78)	164 (21.24)	
Yes	848 (76.47)	240 (71.22)	608 (78.76)	
**Taken naproxyn for pain, n (%)**				< 0.001
No	570 (51.40)	202 (59.94)	368 (47.67)	
Yes	539 (48.60)	135 (40.06)	404 (52.33)	
**Taken indomethacin for pain, n (%)**				0.034
No	1083 (97.66)	334 (99.11)	749 (97.02)	
Yes	26 (2.34)	3 (0.89)	23 (2.98)	
**Taken Cox‐2 inhibitor for pain, n (%)**				< 0.001
No	950 (85.66)	313 (92.88)	637 (82.51)	
Yes	159 (14.34)	24 (7.12)	135 (17.49)	
**Taken aspirin for pain, n (%)**				0.196
No	773 (69.70)	244 (72.40)	529 (68.52)	
Yes	336 (30.30)	93 (27.60)	243 (31.48)	
**The type of analgesic taken, n (%)**				< 0.001
None	173 (15.60)	67 (19.88)	106 (13.73)	
One	300 (27.05)	109 (32.34)	191 (24.74)	
Two or more	636 (57.35)	161 (47.77)	475 (61.53)	
**Sleep duration (h), M (Q_1_, Q_3_)**	7.00 (6.00, 8.00)	7.00 (6.00, 8.00)	6.00 (6.00, 8.00)	0.013
**Sleep group, n (%)**				0.001
< 7 h	543 (48.96)	148 (43.92)	395 (51.17)	
7–9h	540 (48.69)	187 (55.49)	353 (45.73)	
> 9 h	26 (2.34)	2 (0.59)	24 (3.11)	
**Sleep disorder, n (%)**				< 0.001
No	634 (57.17)	233 (69.14)	401 (51.94)	
Yes	475 (42.83)	104 (30.86)	371 (48.06)	

Abbreviations: BMI = body mass index; CP = chronic pain; M = Median; PIR = family income to poverty ratio; Q1 = the first quartile; Q3 = the third quartile; SAP = subacute pain.

Continuous variables with skewness distribution were described as mean (the first quartile, third quartile), and categorical variables were described as numbers (percentage). *P* values were calculated by Mann‐Whitney test and Chi‐square test.

### 3.2 The Impact of Pain on Sleep Disorders

To assess the association between pain characteristics and SD, multivariate logistic regression models were constructed (Table 2). CP demonstrated a significantly higher incidence of SD compared to SAP, with consistent effects across all adjusted models: Model 1 (unadjusted): OR = 2.07 (95% CI, 1.58–2.72; *p* < 0.001); Model 2 (partially adjusted): OR = 1.86 (95% CI, 1.41–2.46; *p* < 0.001); Model 3 (fully adjusted): OR = 1.85 (95% CI, 1.39–2.47; *p* < 0.001). The pain pattern over the day was further stratified into four categories. Compared to individuals with decreasing pain, those in the stay the same group exhibited 47% higher odds of SD (OR = 1.47; 95% CI, 1.04–2.07; *p* = 0.028); those in the increasing group demonstrated nearly twofold higher odds (OR = 1.95; 95% CI, 1.35–2.80; *p* < 0.001). Pain that interfered with sleep was strongly associated with SD: pain woke people from sleep, and conferred a 2.7‐fold increased risk (OR = 2.70; 95% CI, 2.06–3.55; *p* < 0.001) compared to those who did not wake up in pain. Pain unrelieved by exercise was associated with a 59% higher likelihood of SD (OR = 1.59; 95% CI, 1.20–2.11; *p* = 0.001) relative to those reporting pain relief after exercise. Notably, pain pattern during rest/sleep showed the strongest association: individuals with increased pain had a 2.38‐fold increased risk of SD (OR = 2.38; 95% CI, 1.65–3.43; *p* < 0.001) compared to those with decreased pain.

**TABLE 2:** Association between pain‐related information and SD in the pain population.

Variables	Model 1		Model 2		Model 3	
	OR (95%CI)	*P*	OR (95%CI)	*P*	OR (95%CI)	*P*
**Type of pain**						
SAP	Reference		Reference		Reference	
CP	2.07 (1.58 ∼ 2.72)	< 0.001	1.86 (1.41 ∼ 2.46)	< 0.001	1.85 (1.39 ∼ 2.47)	< 0.001
**Pain pattern over the day**						
Decreases	Reference		Reference		Reference	
Stays the same	1.69 (1.22 ∼ 2.33)	0.002	1.67 (1.20 ∼ 2.32)	0.002	1.47 (1.04 ∼ 2.07)	0.028
Increases	2.33 (1.65 ∼ 3.29)	< 0.001	2.22 (1.56 ∼ 3.16)	< 0.001	1.95 (1.35 ∼ 2.80)	< 0.001
It varies, no pattern	1.47 (0.92 ∼ 2.36)	0.108	1.47 (0.91 ∼ 2.37)	0.119	1.31 (0.80 ∼ 2.16)	0.287
**Pain pattern with rest or sleep**						
Decreases	Reference		Reference		Reference	
Stays the same	1.40 (1.03 ∼ 1.91)	0.032	1.41 (1.03 ∼ 1.94)	0.031	1.36 (0.98 ∼ 1.88)	0.065
Increases	2.77 (1.96 ∼ 3.91)	< 0.001	2.61 (1.83 ∼ 3.70)	< 0.001	2.38 (1.65 ∼ 3.43)	< 0.001
It varies, no pattern	1.37 (0.84 ∼ 2.25)	0.209	1.31 (0.79 ∼ 2.17)	0.291	1.34 (0.80 ∼ 2.25)	0.268
Doesn't have rest or sleep pain	0.82 (0.36 ∼ 1.84)	0.629	0.86 (0.38 ∼ 1.99)	0.731	0.87 (0.37 ∼ 2.05)	0.756
**Pain alleviation after exercise**						
Yes	Reference		Reference		Reference	
No	1.84 (1.41 ∼ 2.41)	< 0.001	1.73 (1.32 ∼ 2.27)	< 0.001	1.59 (1.20 ∼ 2.11)	0.001
Does not do these activities	2.04 (1.12 ∼ 3.69)	0.019	1.98 (1.08 ∼ 3.63)	0.028	1.71 (0.91 ∼ 3.20)	0.096
**Pain wakes person from sleep**						
No	Reference		Reference		Reference	
Yes	2.92 (2.28 ∼ 3.74)	< 0.001	2.91 (2.26 ∼ 3.76)	< 0.001	2.70 (2.06 ∼ 3.55)	< 0.001

Model 1, crude.

Model 2, adjusted for sex, age, and race.

Model 3, adjusted for sex, age, race, marital status, education level, hypertension, hyperlipidemia, alcohol status, smoking status, PIR, and BMI.

Abbreviations: BMI = body mass index; CI = confidence interval; CP = chronic pain; OR = odds ratio; PIR = family income to poverty ratio; SAP = subacute pain; SD = sleep disorder.

To further elucidate the impact of analgesics on the correlation between subacute, chronic pain, and sleep disorders (Supplementary Material, T1). Comparative analysis revealed a significantly elevated incidence of sleep disorders among Cox‐2 inhibitor users in both groups, though with divergent effect magnitudes. Notably, the CP cohort exhibited an association (OR, 2.31; 95% CI, 1.52–3.50; *p* < 0.001), and the SAP group also demonstrated a statistically significant relationship (OR, 2.76; 95% CI, 1.09–6.98; *p* = 0.033).

**FIGURE 3:**
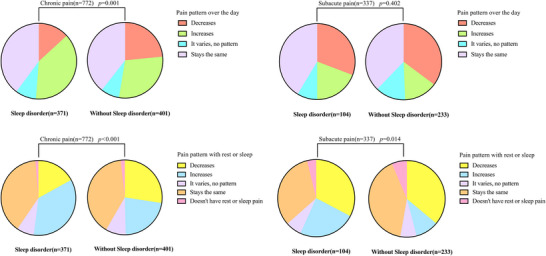
The distribution of pain patterns in the CP and SAP groups stratified by SD. Abbreviations: CP = chronic pain; SAP = subacute pain; SD = sleep disorder.

To further examine the association between pain characteristics and SD in SAP and CP populations, we compared the following variables stratified by SD status: pain pattern over the day, pain pattern with rest or sleep, pain response to exercise, and pain waking the person from sleep. In the CP cohort, significant differences in pain patterns were observed between individuals with and without SD, both throughout the day (*p* = 0.001) and at rest (*p* < 0.001). Similarly, in the SAP cohort, pain patterns differed during rest/sleep based on SD status (*p* < 0.001) (Figure 3). Exercise‐induced pain relief was significantly more prevalent in CP patients without SD than in those with SD (73.57% vs. 57.41%, *p* < 0.001). In contrast, no significant association was observed in the SAP cohort (*p* = 0.197). SD was strongly associated with nocturnal pain‐related awakenings, exhibiting higher prevalence in both CP (69.27% vs. 46.88%, *p* < 0.001) and SAP patients (52.88% vs. 27.04%, *p* < 0.001). (Supplementary material T2)

### 3.3 Subgroup Analyses

**FIGURE 4:**
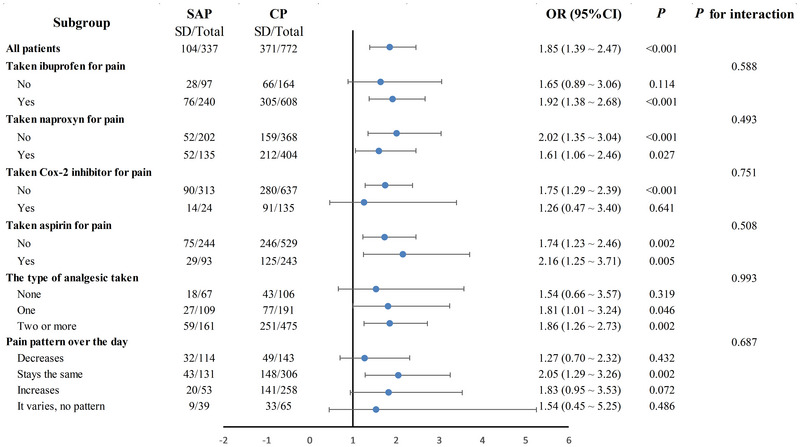
Subgroup analyses between pain and SD. Abbreviations: BMI = body mass index; CI = confidence interval; CP = chronic pain; OR = odds ratio; PIR = family income to poverty ratio; SAP = subacute pain; SD = sleep disorder. The model was adjusted for sex, age, race, marital status, education level, hypertension, hyperlipidemia, alcohol status, smoking status, PIR, and BMI.

Subgroup analyses were performed to assess potential effect modification by analgesic use and pain patterns on the pain–SD association (Figure 4). Notably, no significant interaction effects were observed for either analgesic class or pain patterns (all *p* for interaction > 0.05). Specifically, the association between SAP, CP, and SD development remained consistent regardless of changes in analgesics and pain patterns. (Ibuprofen, *p* for interaction = 0.588; naproxen, *p* for interaction = 0.493; Cox‐2 inhibitor, *p* for interaction = 0.751; aspirin, *p* for interaction = 0.508; the type of analgesic taken, *p* for interaction = 0.993; pain pattern over the day, *p* for interaction = 0.687). These findings suggest that neither pharmacological pain management nor pain pattern significantly moderates the pain‐SD relationship, supporting the robustness of the primary association.

## 4 Discussion

This study demonstrates that individuals with CP exhibit a significantly higher risk of developing SD compared to those with SAP. Key risk factors for SD included unmedicated high pain intensity, ineffective pain relief through exercise, and pain‐related nighttime awakenings. Notably, the use of pain medication and fluctuations in pain intensity did not significantly modify the association between pain chronicity (subacute vs. chronic) and SD development. A robust body of evidence has investigated the association between CP and SD, with numerous studies elucidating their complex bidirectional relationship. This interaction is mediated through several neurophysiological mechanisms. For instance, sleep deprivation impairs the anti‐nociceptive function of endogenous opioids within the central nervous system, resulting in heightened pain sensitivity that exacerbates chronic pain severity. Conversely, chronic pain induces abnormal alterations in central monoaminergic and opioid signaling pathways. These neuroadaptive changes contribute to the central dysregulation implicated in the pathogenesis of sleep disorders, which frequently co‐occur with chronic pain conditions (Haack et al. 2020, Andersen et al. 2018, Menefee et al. 2000). While the association between CP and SD has been extensively documented, research examining SAP remains limited. To our knowledge, this study represents the first investigation utilizing the NHANES database to systematically evaluate the relationship between pain duration (subacute versus chronic) and SD risk. Our findings provide novel insights into the temporal dimension of pain‐related SD, demonstrating a graded association between pain duration and SD risk.

**FIGURE 5:**
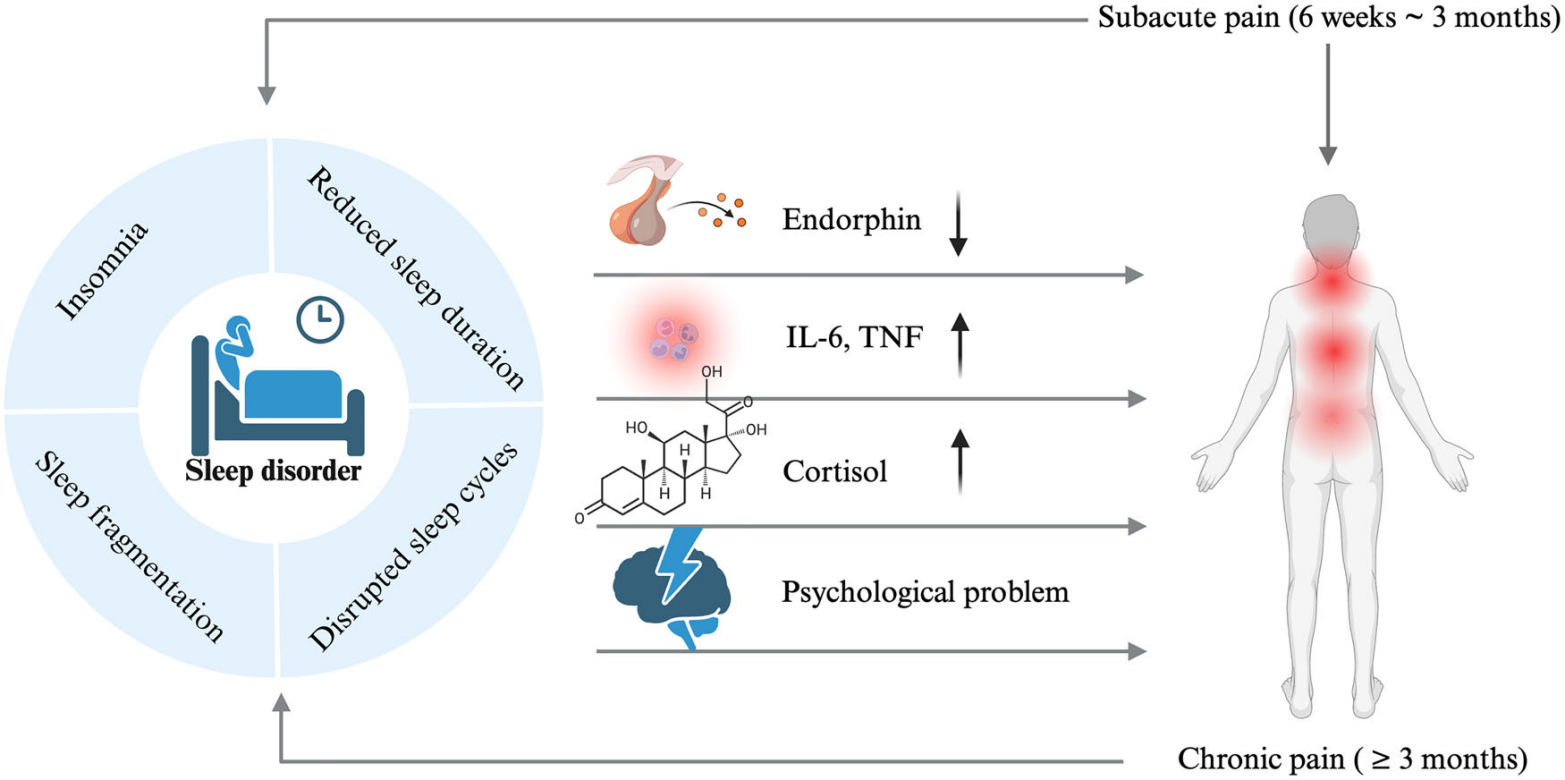
The bidirectional mechanism between CP, SAP, and SD. Abbreviations: CP = chronic pain; SAP = subacute pain; SD = sleep disorder. This image was generated with the Biorender software.

Our analysis revealed a significantly stronger association between CP and SD compared to SAP (OR = 1.85; 95% CI, 1.39–2.47; *p* < 0.001), suggesting that pain duration may be an important modifier in this relationship. SD may contribute to heightened pain sensitivity through multiple pathophysiological mechanisms: impaired endogenous endorphin release, altered opioid receptor expression and function, elevated pro‐inflammatory cytokines (including IL‐6 and TNF‐α), hypothalamic‐pituitary‐adrenal (HPA) axis dysregulation, and neurotransmitter imbalances affecting serotonin, dopamine, and norepinephrine pathways (Vacas et al. 2013, Kassab et al. 2006, Irwin and Opp 2017, Eacret et al. 2023, Husak and Bair 2020). These physiological alterations, compounded by secondary psychological comorbidities, collectively reduce pain thresholds and facilitate central sensitization, thereby creating a vicious cycle of pain‐sleep dysfunction (Figure 5). The SAP phase, representing a critical transitional period between acute and CP states, may constitute an optimal therapeutic window for preventing the development of pain‐related sleep disturbances. Our findings suggest that targeted interventions during this vulnerable period could potentially mitigate the progression to more persistent sleep disorders.

The current study identified exercise‐unalleviated pain as a significant predictor of SD (OR = 1.59; 95% CI, 1.20‐2.11; *p* = 0.001). This finding contrasts with established evidence demonstrating exercise‐induced sleep improvements in fibromyalgia populations (Villafaina et al. 2019, Ericsson et al. 2016). The discrepancy may reflect fundamental differences in pain pathophysiology, as neuropathic CP appears to more profoundly disrupt both nociceptive processing and sleep‐wake regulation compared to non‐neuropathic pain conditions. In this nationally representative analysis, Cox‐2 inhibitor use was significantly associated with an increased risk of SD among patients with pain. The adjusted odds ratios were 2.76 (95% CI, 1.09–6.98; *p* = 0.033) in patients with SAP and 2.31 (95% CI, 1.52–3.50; *p* < 0.001) in those with CP. However, the wide confidence interval observed in the SAP subgroup—likely attributable to limited statistical power (n = 24 Cox‐2 inhibitor users)—suggests that this finding should be interpreted with caution. The smaller sample size in this stratum may have led to unstable estimates, underscoring the need for replication in larger cohorts. Proactive clinical evaluation and early intervention in patients experiencing SAP may reduce the incidence of SD. For individuals with CP, a comprehensive multimodal therapeutic strategy—incorporating pharmacotherapy, physiotherapy, psychological interventions, and lifestyle modifications—is critical for preventing the development or progression of pain‐related SD (Hylands‐White et al. 2017).

This study has several limitations that should be acknowledged. First, due to the cross‐sectional design of NHANES, we cannot infer causal relationships between CP, SAP, and SD. Longitudinal or interventional studies are needed to elucidate potential temporal associations. Second, the assessment of SD relied on self‐reported questionnaire data, which may be subject to recall bias and misclassification. Incorporating objective measures, such as polysomnography or actigraphy, would enhance the validity of sleep‐related findings in future research. Third, the available NHANES data on pain localization (e.g., back, neck, buttocks, hips, and ribs) were limited. The sample size for single‐region pain was particularly restricted, as the dataset also captured multiregional pain. Future investigations with larger, more detailed pain phenotyping could examine whether SD manifestations vary by specific anatomical sites. Fourth, the heterogeneity in analgesic classifications presents an analytical challenge. Grouping non‐steroidal anti‐inflammatory drugs (NSAIDs) with aspirin may obscure important mechanistic distinctions. Furthermore, the NHANES questionnaire does not allow for differentiation between specific types of pain (e.g., osteoarthritis, muscular, or neuropathic), which may have distinct pathophysiological pathways and potentially variable relationships with sleep outcomes. Future studies with more detailed pain phenotyping are warranted to explore these specific associations. Finally, this study highlights the need for further research into the progression of sleep deterioration in SAP groups, as well as the identification of predictive biomarkers for subacute‐to‐chronic pain transition. Such insights could facilitate early, targeted interventions to mitigate SD risk in affected individuals.

## 5 Conclusion

This cross‐sectional study utilized data from the NHANES 2009–2010 to examine the association between SAP, CP, and SD among U.S. adults. Statistical analysis revealed a significantly higher prevalence of SD in individuals with CP compared to those with SAP (*p* < 0.001), suggesting a potential dose‐response relationship between pain duration and SD. This finding underscores the critical significance of early intervention in SAP to mitigate the development of a detrimental pain‐sleep cycle. However, given the inherent limitations of cross‐sectional data from the NHANES, the observed association between CP and SD cannot establish temporal or causal relationships. Future longitudinal studies with prospective designs are warranted to validate these preliminary associations and elucidate potential mechanistic pathways. Proactive sleep monitoring in individuals presenting with SAP may serve as a preventive strategy to attenuate the incidence and severity of SD. While the magnitude of this effect requires further empirical quantification, such interventions could disrupt the maladaptive progression toward CP‐sleep comorbidity.

## Author Contributions

**Yanlin Yang**: conceptualization, software, validation, data curation, formal analysis, methodology, and writing – original draft. **Gaohui Wu**: conceptualization, software, validation, investigation, visualization. **Minxian Wang**: conceptualization, software, validation. **Shuai Zhao**: funding acquisition, project administration, resources, supervision, and writing – review and editing.

## Ethics Statement

Data for this study were gathered from the NHANES public database, which has been authorized by the Ethics Review Board (ERB) as appropriate, and all respondents completed a written informed consent form before engaging in the survey to enter data.

## Peer Review

The peer review history for this article is available at https://publons.com/publon/10.1002/brb3.70976.

## Supporting information




**Supplementary Material**: brb370976‐sup‐0001‐Tables.docx

## Data Availability

All data presented in this study is accessible at the following URL: https://www.cdc.gov/nchs/nhanes/index.html
