# Supplementary material for: The Relationship Between Subacute Pain, Chronic Pain, and Sleep Disorder: A Cross‐Sectional Study Based on NHANES (2009–2010)
Source: Brain Behav. 2025 Oct 21;15(10):e70976. doi: 10.1002/brb3.70976 (PMC12541131; doi:10.1002/brb3.70976)
Supplement: Supplementary file 1 — Supplementary Material: brb370976‐sup‐0001‐Tables.docx [file BRB3-15-e70976-s001.docx]

**T1**. The relationship between analgesics and SD in SAP/CP groups.

Abbreviations: OR: odds ratio; CI: confidence interval; PIR: family income to poverty ratio; BMI: body mass index; CP: chronic pain; SAP: subacute pain; SD: sleep disorder.

The model was adjusted for sex, age, race, marital status, education level, hypertension, hyperlipidemia, alcohol status, smoking status, PIR, and BMI.

| **Analgesic** | **Subacute pain** | | **Chronic pain** | |
| --- | --- | --- | --- | --- |
|  | **OR (95% CI)** | ***P* value** | **OR (95% CI)** | ***P* value** |
| Taken ibuprofen for pain |  |  |  |  |
| No | Reference |  | Reference |  |
| Yes | 1.16 (0.66 ~ 2.03) | 0.602 | 1.37 (0.95 ~ 1.99) | 0.094 |
| Taken naproxyn for pain |  |  |  |  |
| No | Reference |  | Reference |  |
| Yes | 1.53 (0.92 ~ 2.55) | 0.100 | 1.27 (0.94 ~ 1.72) | 0.121 |
| Taken indomethacin for pain |  |  |  |  |
| No | Reference |  | Reference |  |
| Yes | 3.33 (0.28 ~ 40.22) | 0.343 | 2.29 (0.88 ~ 5.98) | 0.091 |
| Taken Cox-2 inhibitor for pain | |  |  |  |
| No | Reference |  | Reference |  |
| Yes | 2.76 (1.09 ~ 6.98) | 0.033 | 2.31 (1.52 ~ 3.50) | <0.001 |
| Taken aspirin for pain |  |  |  |  |
| No | Reference |  | Reference |  |
| Yes | 0.81 (0.46 ~ 1.43) | 0.472 | 1.19 (0.86 ~ 1.65) | 0.295 |

**T2.** The characteristics of pain in the CP and SAP groups.

Abbreviations: CP: chronic pain; SAP: subacute pain; SD: sleep disorder; Non-SD: without sleep disorder.

Categorical variables were described as numbers(percentages). *P* values were calculated by Chi-square test.

| **Variables** | **CP (n = 772)** | | | | **SAP (n = 337)** | | | |
| --- | --- | --- | --- | --- | --- | --- | --- | --- |
|  | **Non-SD (n = 401)** | **SD (n = 371)** | **Statistic** | ***P*** | **Non-SD (n = 233)** | **SD (n = 104)** | **Statistic** | ***P*** |
| **Pain pattern over the day, n(%)** | |  | χ²=15.59 | 0.001 |  |  | χ²=2.93 | 0.402 |
| Decreases | 94 (23.44) | 49 (13.21) |  |  | 82 (35.19) | 32 (30.77) |  |  |
| Increases | 117 (29.18) | 141 (38.01) | |  | 33 (14.16) | 20 (19.23) |  |  |
| It varies, no pattern | 32 (7.98) | 33 (8.89) |  |  | 30 (12.88) | 9 (8.65) |  |  |
| Stays the same | 158 (39.40) | 148 (39.89) | |  | 88 (37.77) | 43 (41.35) |  |  |
| **Pain pattern with rest or sleep, n(%)** | | | χ²=21.16 | <0.001 |  |  | χ²=12.45 | <0.001 |
| Decreases | 110 (27.43) | 63 (16.98) |  |  | 84 (36.05) | 34 (32.69) |  |  |
| Increases | 89 (22.19) | 130 (35.04) | |  | 23 (9.87) | 25 (24.04) |  |  |
| It varies, no pattern | 35 (8.73) | 28 (7.55) |  |  | 16 (6.87) | 7 (6.73) |  |  |
| Stays the same | 160 (39.90) | 145 (39.08) | |  | 95 (40.77) | 34 (32.69) |  |  |
| Doesn't have rest or sleep pain | 7 (1.75) | 5 (1.35) |  |  | 15 (6.44) | 4 (3.85) |  |  |
| **Pain alleviation after exercise, n(%)** | |  | χ²=22.39 | <0.001 |  |  | χ²=3.24 | 0.197 |
| No | 93 (23.19) | 140 (37.74) | |  | 56 (24.03) | 27 (25.96) |  |  |
| Yes | 295 (73.57) | 213 (57.41) | |  | 169 (72.53) | 69 (66.35) |  |  |
| Does not do these activities | 13 (3.24) | 18 (4.85) |  |  | 8 (3.43) | 8 (7.69) |  |  |
| **Pain wakes person from sleep, n(%)** | | | χ²=39.57 | <0.001 |  |  | χ²=21.11 | <0.001 |
| No | 213 (53.12) | 114 (30.73) | |  | 170 (72.96) | 49 (47.12) |  |  |
| Yes | 188 (46.88) | 257 (69.27) | |  | 63 (27.04) | 55 (52.88) |  |  |
